# Supplementary material for: Chromothripsis during telomere crisis is independent of NHEJ, and consistent with a replicative origin
Source: Genome Res. 2019 May;29(5):737–49. doi: 10.1101/gr.240705.118 (PMC6499312; doi:10.1101/gr.240705.118)
Supplement: Supplemental Material [file supp_gr.240705.118_Supplemental_file_1.zip › contigs/annotated_contigs/DB45/contig.2.DB45_length_770_mean_cov_7.25324675325.docx]

**DB45_length_770_mean_cov_7.25324675325**

```
TGTAAGAAGACAGTGGATATGTACATTTAGCTTAAATTAAGTGGAATTGTACCATAGTTTCACATTACTTTTATTGTGTGAGTATGAGA
 >chr3:61581923-61582218 + E=3e-157
CAACATACATATTCATTTATAAAAACAACAGGAATTTTTGCAAGAGAAAAACAGATTTCACTAGTGGTTTTCAAAACACAGAATCTGTT

TCAATTCGGATGGATTTGTTGTTGTTGTTGTTGAAGCACAGTATATGAAACACGTAGCATGACCTCTTTATTTGACAAATAACTGGGGC

AGTGTAGGGCAAGACACTTGCTATCTGAG|GC|AATATCTACAAGGGACTTGTATCTTTCAGACTTTTCAGTAAACAAGTTTCCAACAT
                              >chr3:61431231-61431705 + E=2e-273
GGCAAATTTTCATGTAGGCGTCAGCAGAGATATTTTCTATGAATGCCTAAAATAATCATCACTTTTTCCATGTAGATATTTTCTATGAA

TACCTAAAATAATCATCATTTTAAGCATATTAAATATTATCTTGGAGAGTGTTACAGCTTACACTCAACTAAAGGCCTAGAATGTAGGT

TATAATAAAAATTTTATTAATAAATAAATGAATGAATCATGCCACCGGTTATTGCCTTTACTGTTAACATCCGGGACAACTTTTACTTC

TCTTTATAATCTCCATTGATTCATAACTTCTCTTGGTTCCCTTATCTGTAAAATGGGAGTAGTAATCGTGCCTCATGATTGATTCAGTG

CCGCATTACTGAATCATTGTGAGGATTCAGTAATGTAGCCTATGGAAAGCACCTAACACC
```
